# Cerebral Venous Sinus Thrombosis, Pulmonary Embolism, and Thrombocytopenia After COVID-19 Vaccination in a Taiwanese Man: A Case Report and Literature Review

**DOI:** 10.3389/fneur.2021.738329

**Published:** 2021-09-24

**Authors:** Wei Lin, Chien-An Ko, Yueh-Feng Sung, Yeu-Chin Chen, Jiunn-Tay Lee, Yun-Qian Lin, Yu-Kai Lin

**Affiliations:** ^1^Department of Neurology, Tri-Service General Hospital, National Defense Medical Center, Taipei, Taiwan; ^2^Department of Hematology and Oncology, Tri-Service General Hospital, National Defense Medical Center, Taipei, Taiwan

**Keywords:** COVID-19, vaccine-induced immune thrombotic thrombocytopenia, cerebral venous sinus thrombosis, pulmonary embolism, thrombocytopenia

## Abstract

**Objective:** Coronavirus disease (COVID-19) vaccine-induced immune thrombotic thrombocytopenia (VITT) is a rare but fatal complication observed within 2 weeks of adenovirus-vectored vaccination.

**Case Report:** A 52-year-old male patient, with a family history of autoimmune diseases, presented with a new onset of worsening headache with nausea and vomiting post-vaccination. The patient was diagnosed with VITT based on laboratory findings demonstrating thrombocytopenia, elevated D-dimer, and dural sinus thrombosis identified on neuroimaging. The patient was successfully treated with high-dose immunoglobulin, steroids, and non-heparin anticoagulants, without any neurologic sequelae. Finally, a confirmatory test with anti-platelet factor 4 antibody was strongly positive.

**Conclusion:** Physicians should be vigilant when treating patients presenting with new-onset thunderclap headache, progressive worsening headache, and awakening headache accompanied by nausea or vomiting after vaccination, even if no definite clinical neurological deficits are identified. Emergency laboratory test results for demonstrating elevated D-dimer levels, decreased platelet count, and neuroimaging correlation are integral for diagnosis and must be the standard protocol. Treatment with non-heparin anticoagulants, high-dose intravenous immunoglobulin, and steroids that halt or slow the immune-mediated prothrombotic process should be initiated immediately. Considering the high mortality rate of VITT, treatment should be initiated prior to confirmatory test results.

## Introduction

The coronavirus disease (COVID-19) pandemic, caused by severe acute respiratory syndrome coronavirus type 2 (SARS-CoV-2), has placed a heavy burden on the global healthcare system. This infectious disease may cause systemic multiorgan complications (1). A significant portion of patients could develop neurological complications involving both the central and peripheral systems. Patients may experience mild symptoms including myalgia, dizziness, headache, confusion, or anosmia (2). Serious neurological complications such as cerebral vascular thromboembolism events (3, 4), seizure (2), parainfectious acute disseminated encephalomyelitis, limbic encephalitis (5), and Guillain-Barré syndrome (6) may be encountered as well. The development of vaccines has helped mitigate the global health crisis caused by COVID-19. Vaccines authorized by the European Medicine Agency could be classified as using either mRNA technology or adenovirus vector-based technology. The Taiwanese population is being vaccinated using the ChAdOx1 nCoV-19 (Oxford-AstraZeneca) vaccine. Vaccine-induced immune thrombotic thrombocytopenia (VITT) is a rare complication that occurs after adenovirus-vectored vaccination and may lead to multiple organ thrombosis. The immune-mediated hypercoagulative condition is caused by anti-platelet 4 (P4) antibodies (7). Cases of VITT developing after the ChAdOx1 nCoV-19 vaccine have been reported in several European countries (7–12). In this paper, we report a case of cerebral venous sinus thrombosis, pulmonary embolism, and thrombocytopenia after COVID-19 vaccination that was successfully treated without any systemic sequelae.

## Case Description

A 52 year-old man presented to the emergency department (ED) with acute onset of progressive worsening headache, mainly over the left temporal region, accompanied by nausea and vomiting in the 5 days following COVID-19 vaccination.

The patient had a long history of lymphoma and underwent autologous hematopoietic cell transplantation 9 years earlier, with regular follow-up at the outpatient department. Additional history revealed he had hyperlipidemia and was a hepatitis B carrier. His family history indicated that his mother, two sisters, and daughter had systemic lupus erythematosus and his brother had rheumatoid arthritis. His vital signs at were recorded as follows: blood pressure, 129/90 mmHg; temperature, 35.4°C; heart rate, 67 bpm; respiratory rate, 18 breaths/min; and oxygen saturation, 96%. A score of 9 was recorded on a numerical rating scale (NRS) for assessment of headache. He had received the first dose of the ChAdOx1 nCoV-19 (Oxford-AstraZeneca) vaccine 10 days prior to his admission to the hospital. He did not experience any visual disturbances, seizure attacks, focal weakness, shortness of breath, or abdominal pain. No petechiae were recorded over his extremities, and neurologic examination demonstrated the absence of focal neurologic deficits. Laboratory test results revealed an elevated D-dimer level of >20 mg/L, thrombocytopenia (platelet count, 99,000/uL; used to be 182,000/uL), and negative naso-oropharyngeal swab for SARS-CoV-2 nucleic acid amplification.

## Diagnostic Assessment

**Figure 1:**
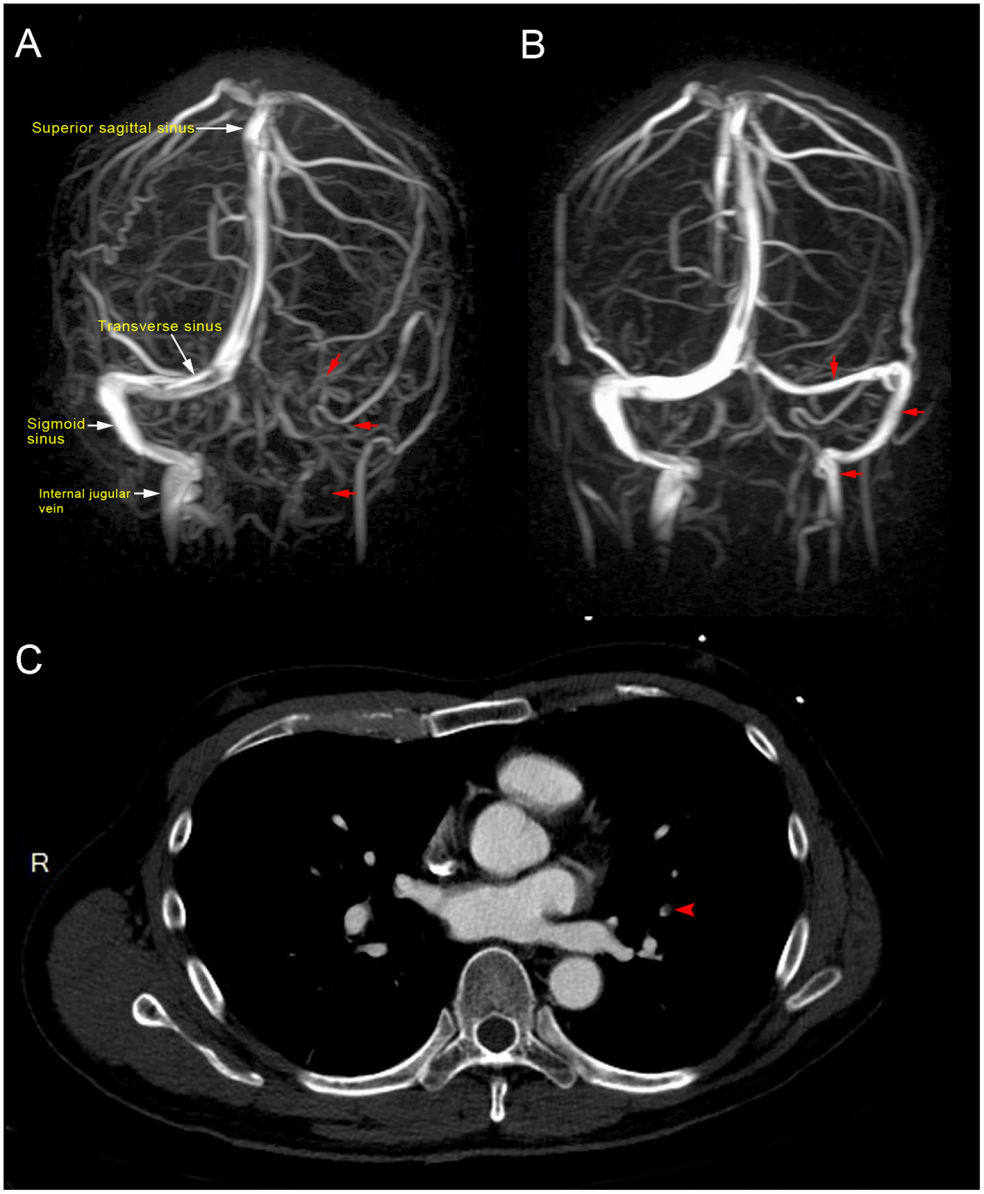
**(A)** Magnetic resonance venography showed loss of signal in the left transverse sinus, sigmoid sinus, and left distal internal jugular vein (red arrow), which were consistent with dural venous sinus thrombosis at 10 days post-vaccination. **(B)**. Magnetic resonance venography after 2 months of direct oral anticoagulant (10 mg apixaban twice daily for initial 7 days, 5 mg twice daily for the following 2 months) showed patent left transverse sinus, sigmoid sinus, and left distal internal jugular vein (red arrow), indicative of recanalization and disease regression. **(C)** Computed tomography showed a filling defect in the left upper lung (red arrow), consistent with pulmonary embolism.

Magnetic resonance imaging (MRI) of the brain showed dural sinus thrombosis in the left transverse sinus, sigmoid sinus, and left distal internal jugular vein (Figure 1A). Chest computed tomography (CT) scan showed filling defects in the left upper lobe, suggesting pulmonary embolism (Figure 1C). Abdominal CT revealed the absence of thrombosis in the abdomen.

**Figure 2:**
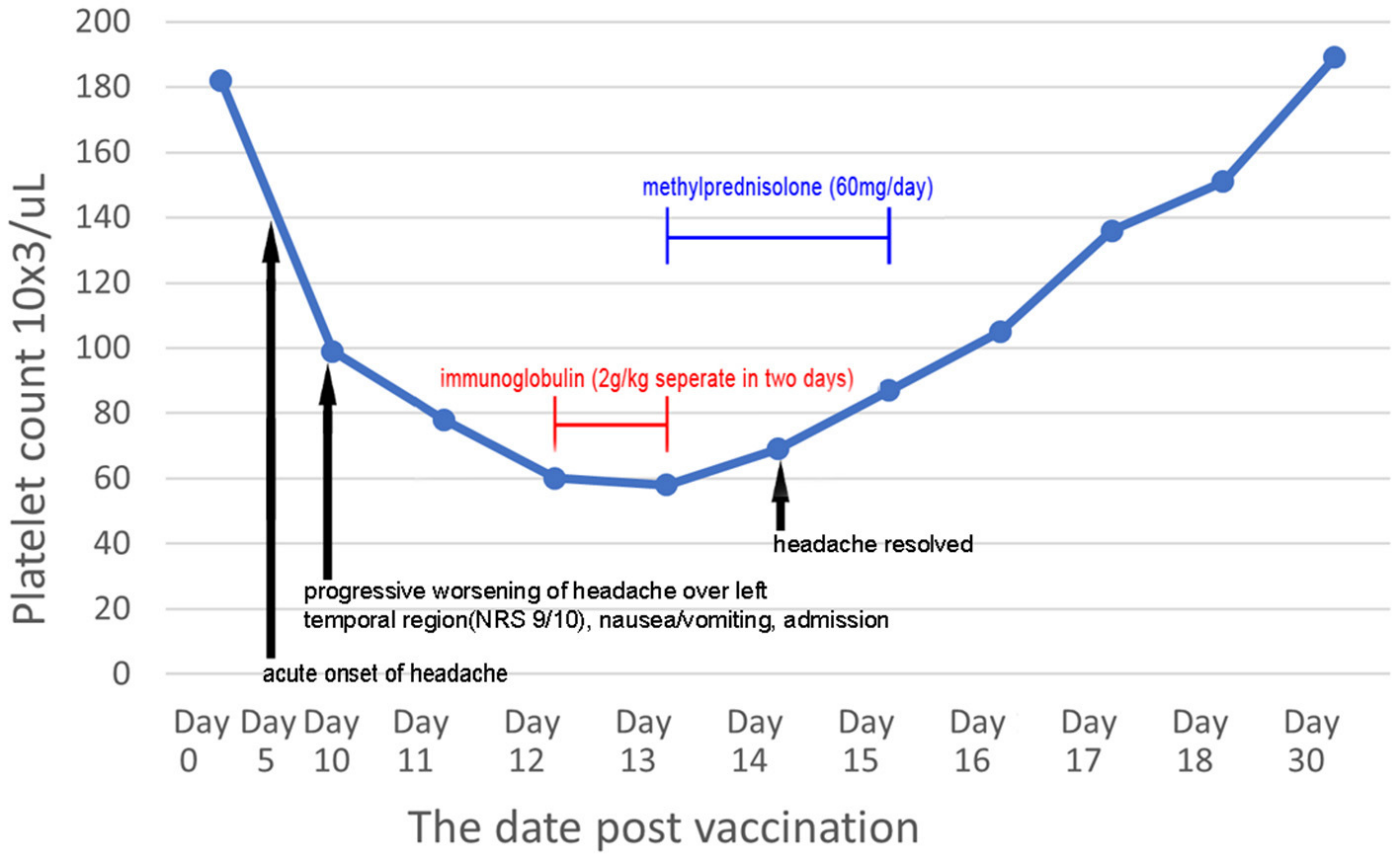
Serial serum platelet count post-vaccination and chronological evolution of symptoms after steroid treatment and high-dose immunoglobulin.

The patient was diagnosed with VITT based on the findings. Treatment was initiated with a direct oral anticoagulant (DOAC; 10 mg apixaban twice daily) to treat cerebral venous thrombosis and pulmonary embolism. An osmotic agent with mannitol (100 mL) was administered twice daily for treatment of severe nausea and vomiting, which indicated increased intracranial pressure. Several serological examinations were conducted to exclude thrombotic thrombocytopenia purpura, paroxysmal nocturnal hemoglobinuria, systemic lupus erythematosus, rheumatoid arthritis, antiphospholipid syndrome, hyperhomocysteinemia, protein C and S deficiency, antithrombin III deficiency, and underlying hematologic malignancies as possible differential diagnoses. Laboratory test results revealed the following: hemoglobin, 13.3 g/dL (13.5–18.0 g/dL); fibrinogen, 283.2 mg/dL (200–400 mg/dL); international normalized ratio, 1.04; lactate dehydrogenase, 153 U/L (140–271 U/L); haptoglobin, 132.4 mg/dL (44.0–215.0 mg/dL); antithrombin III, 91.9% (75–125%); protein C, 100.1% (70–140%); protein S, 85.2% (72.2–126.0%); ADAMTS13, 87.2% (40–130%); lupus anticoagulant, 0.96 (<1.20); rheumatoid factor, <10 IU/mL (<14.0 IU/mL); C3, 96.2 mg/dL (87.0–200.0 mg/dL); C4, 31.1 mg/dL (19.0–52.0 mg/dL); anti-dsDNA, <0.5 IU/mL (<15 IU/mL); negative ANA titer; Anti-Ro, anti-La <0.3 U/mL (<10.0 U/mL); anti-β2 glycoprotein 1 <0.6 U/mL (<10 U/mL); anti-cardiolipin IgG <0.5 U/mL (<40 IU/mL); homocysteine, 10.10 μmol/L (5.0–15.0 μmol/L); anti-myeloperoxidase <0.2 IU/mL (<5.0 IU/mL), anti-proteinase 3 antibody <0.2 IU/mL (<5.0 IU/mL); and decreased platelet count from a peripheral blood smear without schistocytes. Following progressive deterioration of the platelet count (drop from 99,000/uL to 60,000/uL), high-dose immunoglobulin (2 g/kg) therapy was administered over a duration of 2 days. However, a confirmatory test for anti-PF4 antibody later showed >200 (reference optical density value 3.055), without previous exposure to heparin. The platelet count, D-dimer level, and the patient's clinical symptoms gradually improved (Figure 2); he was discharged, without sequelae, 10 days after admission to the hospital. Following brain magnetic resonance venography after DOAC usage for 2 months (10 mg apixaban twice daily for initial 7 days, 5 mg twice daily for the following 2 months) showed patent left transverse sinus, sigmoid sinus, and left distal internal jugular vein, consistent with recanalization and disease regression (Figure 1B). No focal neurological deficits were identified at the outpatient department in follow-up.

## Discussion

COVID-19 VITT is a rare, catastrophic hypercoagulative condition following vaccination that may lead to thrombosis and thrombocytopenia. Thrombosis occurring in the cerebral veins, splanchnic system, portal and hepatic veins, pulmonary veins, cerebral artery, and other systemic sites have been reported (7–13). Based on the available literature, VITT is most commonly observed in Caucasians below the age of 60 years, with no history of previous thrombotic events or family history of thrombophilia, and predominantly in females. Symptoms appear 5–20 days after administration of the AstraZeneca COVID-19 vaccine or Ad26.COV2.S COVID-19 (Janssen/Johnson & Johnson) vaccine, with mortality rates up to 54.6% (8). Patients reported headaches, nausea/vomiting, dizziness, visual disturbances, seizure attacks, focal neurological signs, shortness of breath, chest pain, abdominal pain, pain in the extremities, or swelling as initial presentations. To date, no thrombotic events and thrombocytopenia associated with authorized mRNA COVID-19 vaccines have been reported to the Vaccine Adverse Event Reporting System (13).

In our case, a Taiwanese man with a family history of autoimmune disease presented with new onset of worsening headache. Signs of potential increased intracranial pressure were recorded 10 days after he received the AstraZeneca COVID-19 vaccine.

Laboratory test results confirmed thrombocytopenia, decreased or normal levels of fibrinogen, and elevated D-dimer levels. Additional diagnostic tools included a positive heparin-induced thrombocytopenia enzyme-linked immunosorbent assay, which identifies anti-PF4 antibody, or functional heparin-induced platelet activation assay or serotonin-release assay (7, 8). Imaging techniques for the diagnosis of VIIT include CT arteriography and venography, magnetic resonance angiography and venography, and arteriography to identify blood clots within organs. In this case, the patient's CT venography and magnetic resonance venography demonstrated thrombosis in the left transverse sinus, sigmoid sinus, and distal internal jugular vein. Thorough tests for autoimmune diseases, thrombophilia, and screening for hypercoagulative conditions such as thrombotic thrombocytopenia purpura, paroxysmal nocturnal hemoglobinuria, systemic lupus erythematosus, rheumatoid arthritis, antiphospholipid syndrome, hyperhomocysteinemia, protein C and S deficiency, antithrombin III deficiency, and underlying hematologic malignancies revealed negative findings.

VITT is an immune-mediated disorder characterized by a hypercoagulative tendency (14). The pathophysiology of VITT is similar to that of heparin-induced thrombocytopenia, a progressive prothrombotic condition, provoked by platelet-activating antibodies that recognize multimolecular complexes between cationic PF4 and anionic heparin, which leads to platelet activation (15). This prothrombotic condition is triggered by highly sulfated and highly negatively charged oligosaccharides, such as pentosan polysulfate, hypersulfated chondroitin sulfate, and related molecules, and the vector of the AstraZeneca vaccine, known as chimpanzee adenovirus, other than heparin (14, 16). Platelet activation eventually results in inflammatory processes, mediated by neutrophils, myeloperoxidase, and lysozymes (17). Management of VITT includes administration of non-heparin anticoagulant agents, danaparoid, argatroban, and direct oral anticoagulants (DOACs), such as apixaban and rivaroxaban (8). High-dose intravenous immunoglobulins (IVIG), with or without steroids, may inhibit platelet-activating antibodies and de-escalate the prothrombotic mechanism (18). Platelet transfusion should be avoided. Vitamin K antagonists are usually contraindicated for thrombocytopenia and disseminated intravascular coagulation. In this case, we prescribed DOAC with 10 mg apixaban twice daily for the first 7 days, followed by 5 mg twice daily for 2 months, after diagnosis of VITT. IVIG and steroids were administered on the second day after admission. The follow-up platelet count revealed a gradual improvement, and no further systemic thrombosis, disseminated intravascular coagulation, intracerebral hemorrhage, or extracranial organ hemorrhage was observed during hospitalization.

COVID-19 VITT is a rare and potentially life-threatening disease that is mediated by platelet-activating antibodies against PF4 causing intracerebral and systemic multi-organ thrombosis and thrombocytopenia. Physicians should be vigilant and well-versed with the presentation of VITT in patients. Recognizing the preliminary signs (new-onset thunderclap headache, progressive worsening headache, and awakening headache accompanied by nausea or vomiting), prompt diagnoses, and provision of suitable treatment are crucial at a time when the world is still adjusting to the after-effects of the COVID-19 pandemic. Specific treatment such as non-heparin anticoagulants, high-dose immunoglobulin, and steroids should be administered before confirmatory test results are obtained to prevent fatal complications.

## Data Availability Statement

The original contributions presented in the study are included in the article/supplementary material, further inquiries can be directed to the corresponding author/s.

## Ethics Statement

Written informed consent was obtained from the individual(s) for the publication of any potentially identifiable images or data included in this article.

## Author Contributions

WL collected the data and prepared the manuscript. Y-KL revised the manuscript. All authors contributed to the article and approved the submitted version.

## Conflict of Interest

The authors declare that the research was conducted in the absence of any commercial or financial relationships that could be construed as a potential conflict of interest.

## Publisher's Note

All claims expressed in this article are solely those of the authors and do not necessarily represent those of their affiliated organizations, or those of the publisher, the editors and the reviewers. Any product that may be evaluated in this article, or claim that may be made by its manufacturer, is not guaranteed or endorsed by the publisher.

## Abbreviations

IVIG: intravenous immunoglobulins
DOAC: direct oral anticoagulants
VAERS: Vaccine Adverse Event Reporting System.
